# Anticandidal and *In vitro* Anti-Proliferative Activity of Sonochemically synthesized Indium Tin Oxide Nanoparticles

**DOI:** 10.1038/s41598-020-60295-w

**Published:** 2020-02-24

**Authors:** Suriya Rehman, Sarah Mousa Asiri, Firdos Alam Khan, B. Rabindran Jermy, Vijaya Ravinayagam, Zainab Alsalem, Reem Al Jindan, Ahsanulhaq Qurashi

**Affiliations:** 1 Department of Epidemic Diseases Research, Institute for Research & Medical Consultations, (IRMC), Imam Abdulrahman Bin Faisal University, Dammam, 31441 Saudi Arabia; 2 Department of Biophysics, Institute for Research & Medical Consultations, (IRMC), Imam Abdulrahman Bin Faisal University, Dammam, 31441 Saudi Arabia; 3 Department of Stem Cell Research, Institute for Research & Medical Consultations, (IRMC), Imam Abdulrahman Bin Faisal University, Dammam, 31441 Saudi Arabia; 4 Department of Nano-Medicine Research, Institute for Research & Medical Consultations, (IRMC), Imam Abdulrahman Bin Faisal University, Dammam, 31441 Saudi Arabia; 5 Department of Microbiology, College of medicine, Imam Abdulrahman Bin Faisal University, Dammam, 31441 Saudi Arabia; 6 Center of excellence in nanotechnology, King Fahd University of petroleum and Minerals Dhahran 31261 Saudi Arabia and Department of Chemistry, Khalifa University of Science and Technology, Main Campus, Abu Dhabi, P.O. Box 127788, United Arab Emirates

**Keywords:** Nanoparticles, Nanoparticles

## Abstract

The present work demonstrates the synthesis, characterization and biological activities of different concentrations of tin doped indium oxide nanoparticles (Sn doped In_2_O_3_ NPs), i.e., (Sn/In = 5%, 10% and 15%). We have synthesized different size (38.11 nm, 18.46 nm and 10.21 nm) of Sn doped In_2_O_3_ NPs. by using an ultra-sonication process. The Sn doped In_2_O_3_ NPs were characterized by by x-ray diffraction (XRD), scanning electron microscopy (SEM), and transmission electron microscopy (TEM) which confirmed the successful doping of tin (Sn) with Indium oxide (In_2_O_3_). Anticandidal activity was performed by standard agar dilution method using *Candida albicans* for the study. The minimum inhibitory/fungicidal concentration (MIC/MFC) values recorded were, 8 & >8 mg/ml for pure In_2_O_3_ NPs, 4 & 8 mg/ml for 5%, 2 & 8 mg/ml for 10%, whereas 1 & >4 mg/ml for 15% Sn doped In_2_O_3_ NPs, respectively. The topographical alteration caused by Sn doped In_2_O_3_ NPs on *Candida* cells, was clearly observed by SEM examination. A significant enhancement in anticandidal activity was seen, when *Candida* cells were exposed to (Sn/In = 5%, 10% and 15%). Moreover, we have also evaluated the impact of Sn-In_2_O_3_ NPs on human colorectal carcinoma cells (HCT-116). The results demonstrated that Sn-In_2_O_3_ NPs (Sn/In = 5%, 10% and 15%), caused dose dependent decrease in the cancer cell viability as the low dosage (2.0 mg/mL) showed 62.11% cell viability, while 4.0, 8.0, 16.0, 32.0 mg/mL dosages showed 20.45%, 18.25%, 16.58%, and 15.58% cell viability. In addition, the treatment of Sn-In_2_O_3_ NPs also showed significant cellular and anatomical changes in cancer cells as examined by microscopes. We have also examined the impact of Sn-In_2_O_3_ NPs (5%, 10%, 15%) on normal cells (HEK-293) and the results demonstrate that Sn-In_2_O_3_ NPs did not reduce the cell viability of normal cells.

## Introduction

The increasing population of world, raises new health challenges, and among them are the increased incidence of infectious diseases and cancer^1^. It is a well-known fact, that fungal infections are an established threat in medicine these days. Among them, genus *Candida*, dominates by its prevalence and increasing influence on humans. Approximately, 50–60% of the hospital acquired infection is *Candida* infection called as candidemia, which is a bloodstream infection with high rates of morbidity and mortality^2^. Such nosocomial infections are becoming a huge challenge, hence its necessary to develop new antibiotic therapeutics, especially based on nanoparticles (NPs). In recent years, metal oxide NPs have been studied broadly for their alluring characteristics, which makes them distinct from their corresponding bulk size material^3^. The NPs have been utilized in the preparation of drugs, detection of protein and pathogens, treatment of different cancers, separation and purification of biological molecules and cells^4^. The main reason for considering NPs, as an effective and alternative therapeutics is that, it can help in preventing the drug resistance. The unchecked use of antibiotics, has resulted in the emergence of several health hazards, like extended drug resistant superbugs^5^. To combat the drug resistance, there is a need to search and modulate new therapeutics as antimicrobials and anticancer agents. Therefore, NPs have offered a potential solution to this problem^2,6^.

Indium oxide nanoparticles (In_2_O_3_) is an essential and interesting nanomaterial for a number of applications, including solar cells, photocatalysts, organic light emitting diodes, architectural glasses, panel displays, etc.^7–9^. Number of studies on the synthesis of different structured In_2_O_3_ like nanotubes, nanowires, nanobelts, nanofibers, have been reported for wide applications^10^. Although, there is no information on In_2_O_3_ as antimicrobial agent to best of our knowledge. Sn is reported to possess antimicrobial activities and has been widely used as a promising dopant with oxides like, In_2_O_3_ and ZnO, for enhancing the antimicrobial, electrical, optical and structural properties^11–14^. There are several reports which suggested that ITO possess toxicity action on the cells and organs^15–20^. However, the database on deep and persistent toxicity, carcinogenicity, genotoxicity, reproductive toxicity, besides skin or eye irritation and sensitization is very inadequate or even missing. In our study, we have made an attempt to study the impact of tin (Sn) doped indium oxide (Sn-In203) nanoparticles (NPs) on human colon cancer cells (HCT-116). Sn is one of the important metals often investigated, as its doping is known to increase the carrier lifetime^21^. Different synthetic approaches have been used for the preparation of Sn-In_2_O_3_ nanostructure, like chemical vapor deposition, calcinations, pulsed laser deposition, reactive thermal deposition and sol–gel process^22^. The synthesis of Sn-In_2_O_3_ NPs by wet chemistry techniques shows an effective control over the morphology, crystallinity and size of the particles. In recent years, sonochemical reaction has become one of the most important wet chemistry method, applied for preparation of ultrafine nano-structured materials^12^.

In the current study, we have synthesized different percentage of Sn doped In_2_O_3_ NPs (Sn/In = 5%, 10% and 15%) by a sonication method. To the best of our knowledge, the study of impact of Sn content over indium oxide on the biological properties has not been reported so far. The present study investigates the effect of Sn doping on structural properties by X-ray diffraction XRD, TEM, and TEM. Further, biological activities of the materials were assessed by using both plate assays and morphological analysis by microscopy.

## Results

### Synthesis and characterization of Sn doped In_2_O_3_ NPs

#### X-ray analysis

**Figure 1:**
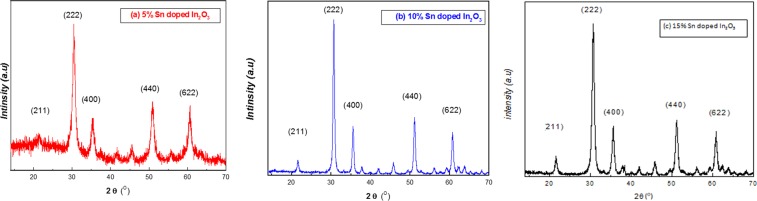
XRD pattern of (**a**) 5% (**b**) 10% and (**c**)15% Sn doped indium oxide NPs.

Fig. 1(a–c) illustrates the XRD of 5%, 10% and 15% Sn doped In_2_O_3_ NPs. Pure In_2_O_3_ NPs were prepared and XRD characterization was analyzed in our previous study. The planes of In_2_O_3_ (211), (222), (400), (440) and (622) showed at 21.39°, 30.89°, 35.59°, 51.45°, and 60.72°, respectively^12^. The peak positions of XRD patterns of 10% and 15% Sn doped In_2_O_3_ NPs, demonstrated the characteristic reflections indexed to the standard of a cubic lattice of In_2_O_3_ (JCPDS No. 06-0416)^23^. The main planes (211), (222), (400), (440) and (622) were observed at 21.7°, 30.8°, 35.7°, 51.9°, and 60.88° for 5% of Sn doped In_2_O_3_ NPs. However, the peaks were found to shift slightly with increase in Sn concentration (10%) to 21.66°, 30.72°, 35.59°, 51°, and 60.76°, while shift was more pronounced with high concentration doping of Sn (15%) to be 21.4°, 30.49°, 35.36°, 50.9°, and 60.58°, respectively. This can be due to the decrease in the lattice parameter, where we see replacement of host ion (In) with larger ionic radii (.81 Å) by dopant Sn ions with smaller radii (71 Å). We have observed high crystalline order without any impurity phase; this is evident from sharp and strong diffraction peaks. The average value of lattice constant *a* was determined by different Miller indices (*hkl*) planes and inter-planer distance *d* by using the formula^24,25^.

1 $$a=d\sqrt{{h}^{2}+{k}^{2}+{l}^{2}}$$

The average crystallite size (*D*) was calculated by Scherer’s equation^26^_._

2 $$D=\frac{{\rm{K}}{\rm{\lambda }}}{{\rm{\beta }}\,\cos \,{\rm{\theta }}}$$

where K is the Scherer constant (0.9), λ is the wavelength (1.5406 nm), θ is the diffraction angle, β is the full width at half maximum (FWHM). The values of lattice parameter *a* and crystallite size *D*, were calculated from high intensity (222) plane with respect to Sn proportion and tabulated in Table 1. The lattice parameter *a* was found at10.15 Å, 10.08 Å and 10.04 Å for 5%, 10% and 15% for Sn doped In oxide, respectively, which shows an decreasing trend with increase of Sn construction.

**Table 1:** *d-*spacing, lattice parameter *a* and crystallite size *D* of Sn doped indium oxide NPs.

Sample	*d-*spacing (Å)	*a* (Å)	*D* (nm)
5% Sn doped In_2_O_3_	2.93	10.15	38.11
10% Sn doped In_2_O_3_	2.91	10.08	18.46
15% Sn doped In_2_O_3_	2.90	10.04	10.21

#### Morphological analysis

**Figure 2:**
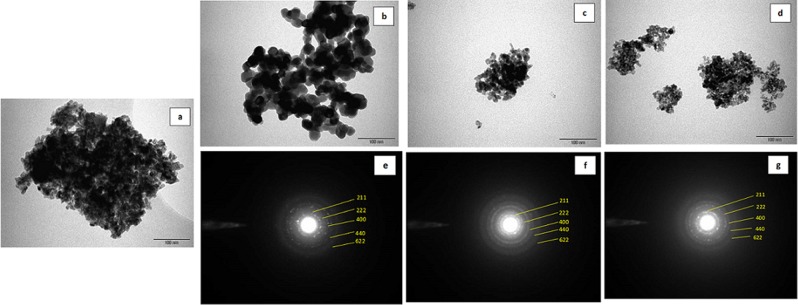
TEM images of (**a**) In_2_O_3_ NPs, (**b**) 5%, (**c**) 10%, and (**d**) 15% Sn doped indium oxide NPs; and selected area electron diffraction (SAED) pattern of (**e**) 5% (**f**) 10% and (**g**) 15% Sn doped indium oxide NPs.

**Figure 3:**
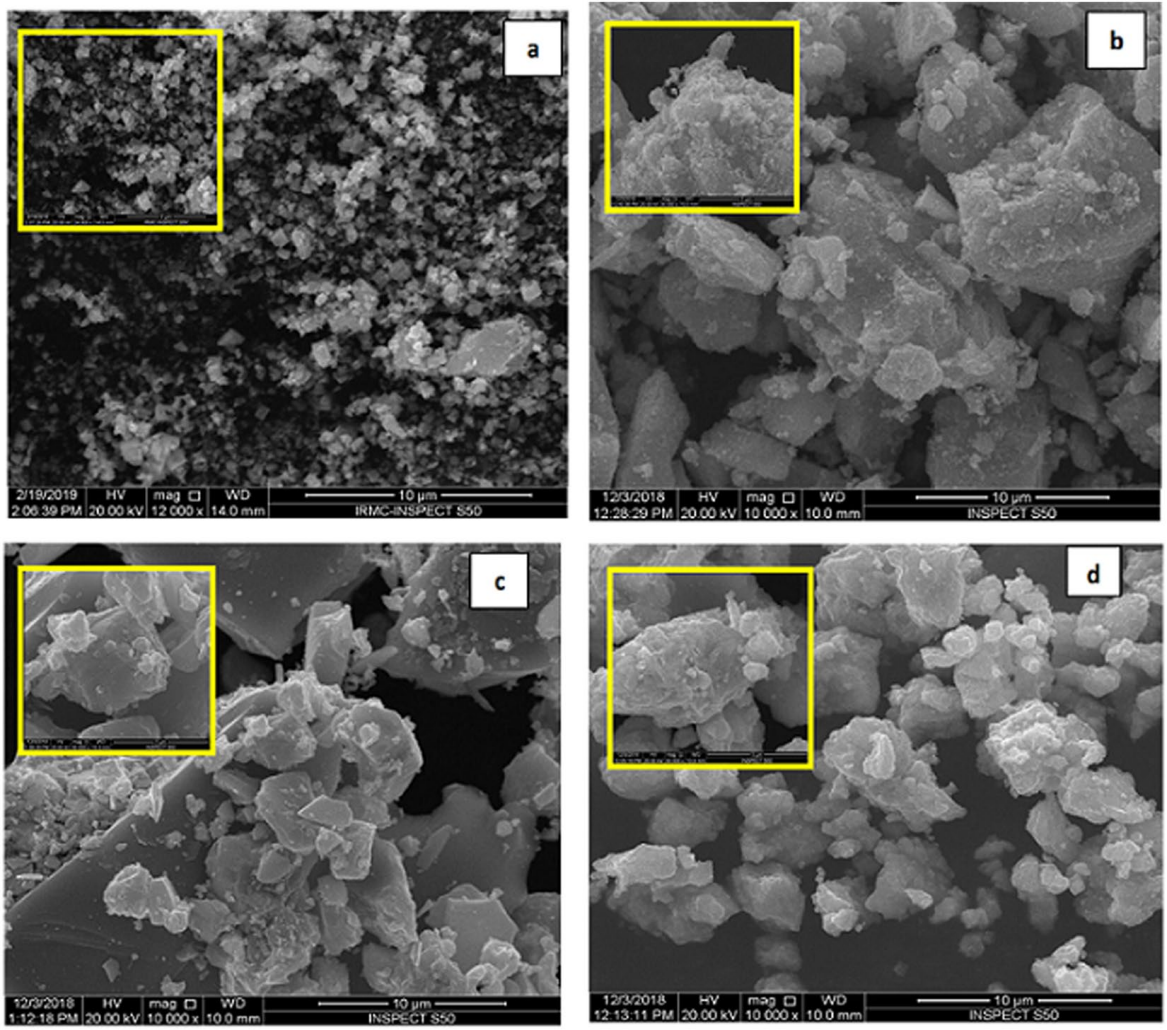
SEM images of (**a**) In_2_O_3_ NPs, (**b**) 5%, (**c**) 10%, and (**d**) 15% Sn doped indium oxide NPs.

Fig. 2(a–d) presents, TEM images of pure In_2_O_3_ NPs and Sn doped In_2_O_3_ NPs (Sn/In = 5%, 10%, 15%). TEM micrographs shows the prepared NPs are irregular in shape and with almost uniform distribution. It was seen that the particle size decreased with the increasing concentration of dopant. Crystallite sizes noted from XRD correlates very well with the results of TEM. The high crystalline order of the samples was further confirmed by selected area electron diffraction (SAED) pattern presented in (Fig. 2(e–g)), where we witnessed the presence of diffused rings, indexed to body-centered cubic In_2_O_3_^27^. Figure 3, on the other hand, depicts SEM morphology with low and high magnification. For In_2_O_3_ NPs, small particles appeared separately, while the surface features of the Sn doped In_2_O_3_ NPs samples showed clearly more agglomeration of particles with different shapes and sizes compared with a pure In_2_O_3_ NPs.

### Anticandidal activity of pure In_2_O_3_ NPs and Sn doped In_2_O_3_ NPs

**Figure 4:**
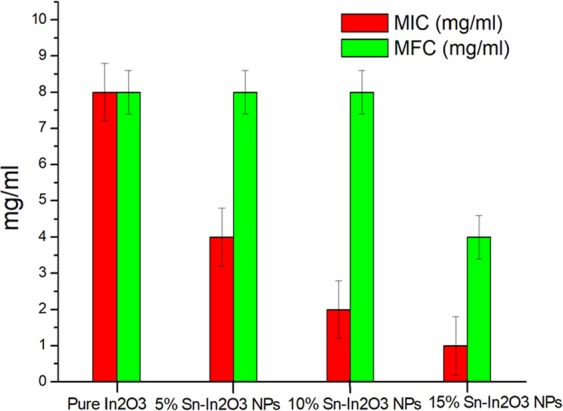
MIC/MFC of pure In_2_O_3_ NPs and Sn doped In_2_O_3_ NPs (Sn/In = 5%, 10%, 15%) against *C. albicans*.

In the present study, anticandidal activity of pure In_2_O_3_ NPs and Sn doped In_2_O_3_ NPs (Sn/In = 5%, 10%, 15%) against *C. albicans*, was evaluated by determining MIC and MFC. The MIC and MFC values of 5%, 10% and 15% Sn doped In_2_O_3_ NPs are summarized in Fig. 4. The MIC and MFC values, obtained were 8 and >8 mg/ml for pure In_2_O_3_ NPs, 4 and 8 mg/ml for 5% Sn doped In_2_O_3_ NPs, 10% Sn doped In_2_O_3_ NPs showed MIC/MFC values of 2 and 8 mg/ml, whereas 15% Sn doped In_2_O_3_ NPs recorded 1 and >4 mg/ml of MIC/MFC, respectively. The anticandidal activity was found in the order of Sn doped, 15% >10% >5% > undoped In_2_O_3_ NPs and the strongest activity was achieved with 15% Sn doped In_2_O_3_ NPs.

### Study of effect of Sn doped In_2_O_3_ NPs on the topology of *C. albicans* by SEM analysis

**Figure 5:**
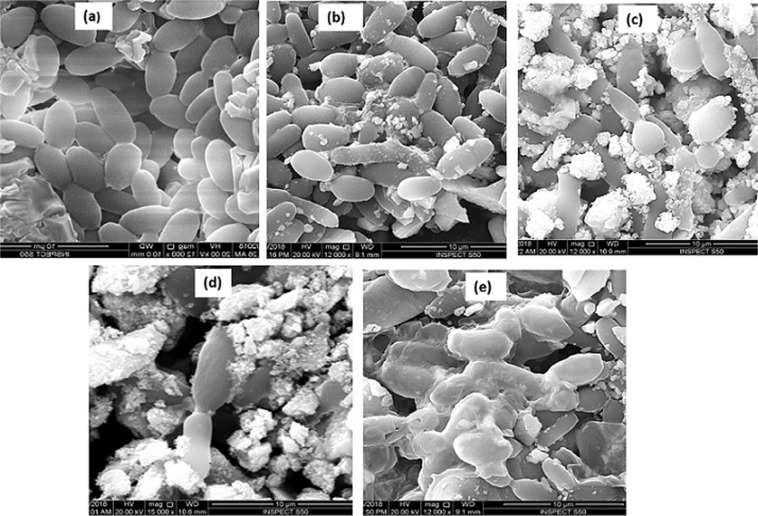
SEM micrographs of *C. albicans (****a****)* untreated (control): treated with (**b**) pure In_2_O_3_ NPs, (**c**) 5%, (**d**) 10%, and (**e**) 15% Sn doped indium oxide NPs.

The morphogenesis caused by Sn doped In_2_O_3_ NPs, were examined by SEM. The untreated *Candida* cells (control), were normal in shape, intact with regular and smooth cell surface (Fig. 5a). However, the treated cells were found to be no more intact and cells were irregular in appearance (Fig. 5). The cells treated with pure In_2_O_3_ NPs and 5% Sn doped In_2_O_3_ NPs, were moderately affected and altered in cell morphology (Fig. 5b,c). However, *Candida* cells treated with 10% Sn doped In_2_O_3_ NPs showed significant alteration, because of the enhanced attachment of NPs to the cell surface (Fig. 5d,e). Further, it has been observed, that the treatment of cells with 15% Sn doped In_2_O_3_ NPs displayed severely damaged cells, due to the profuse attachment and penetration, which led to the formation of pits. Deformation and distortion of cell wall and membrane can be clearly seen in Fig. 5e, indicating loss of membrane integrity, that could possibly cause the cell death.

### Study on hyphal growth in liquid medium and biofilm study of treated *C. albicans* by SEM has also been carried out. The details are included in supplementary file

#### In vitro antiproliferative activity of Sn-In_2_O_3_ NPs on cancerous cells (HCT-116)

**Figure 6:**
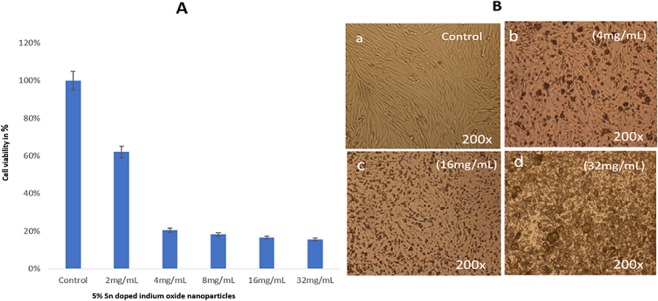
(**A**) MTT Assay (**B**) Cell morphology of HCT-116 cells after treatment with different concentrations of 5% Sn doped indium oxide at 200 × magnifications.

The effect of Sn-In_2_O_3_ NPs on cancerous cells was examined by both morphological analysis and MTT assay. Post 48 h treatments, we have found that (5%) Sn-In_2_O_3_ NPs induced dose-dependent response on the cancer cells as the low dosage **(**2.0 mg/mL) showed 62.11% cell viability (Fig. 6A), while 4.0, 8.0, 16.0, 32.0 mg/mL dosages reduced the cancer cell viability to 20.45%, 18.25%, 16.58%, and 15.58% respectively. With a view to examine the morphologically changes in cancer cells, we have found that Sn-In_2_O_3_ NPs (5%) treated cancer cells showed moderate impact on cancer cells membrane and nucleus (Fig. 6B) with compare to control group cells (Fig. 6Ba). The treatment of 4.0 mg/mL has shown significant impact on cancer cell morphology, such as the cell membrane disruption, and nuclear condensation (Fig. 6B,Ba). The treatment of higher dosages (16.0 mg/mL and 32.0 mg/mL) showed most significant effect on the cancer viability and morphology (Fig. 6B–d).

**Figure 7:**
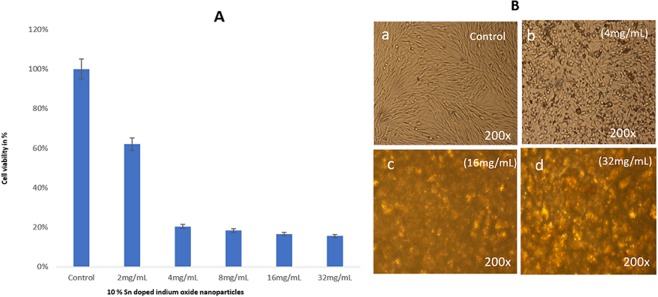
(**A**) MTT Assay (**B**) Cell morphology of HCT-116 cells after treatment with different concentrations of 10% Sn doped indium oxide at 200 × magnifications.

The treatment of 10% Sn-In_2_O_3_ NPs has shown a dose-dependent activity on cancer cells. We have found that the dose **(**2.0 mg/mL) showed 79.05% cell viability (Fig. 7A), while dosages of 4.0, 8.0, 16.0 & 32.0 mg/m showed 46.94%, 30.25%, 29.05%, and 28.75% cancer cell viability. The cell morphology of Sn-In_2_O_3_ NPs treated cancer cells were observed microscopically (7B). No change is observed in the morphology of control group cells (Fig. 7Ba). The dose 4.0 mg/mL showed cell membrane disruption, nuclear condensation (Fig. 7Bb,Ba), while 16.0 mg/mL and 32.0 mg/mL respectively showed strong disintegration, condensation fragmentation of the nucleus (Fig. 7Bc,Bd).

**Figure 8:**
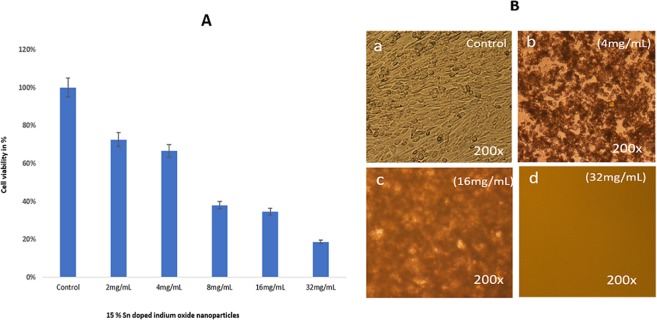
(**A**) MTT Assay (**B**) Cell morphology of HCT-116 cells after treatment with different concentrations of **1**5% Sn doped indium oxide at 200 × magnifications.

The cells, when treated with Sn-In_2_O_3_ NPs (15%), the dose **(**2.0 mg/mL), showed only 72.16% cell survivability (Fig. 8); while dosages of 4.0, 8.0, 16.0 & 32.0 mg/mL, showed decrease of 66.66%, 37.94%, 34.94%, and 18.58%, respectively, in cancer cell viability (Fig. 8). The cell morphology of Sn-In_2_O_3_ NPs treated cells were observed under microscope. We did not find any change in cancer cell morphology in control group cells (Fig. 8Ba). The dose of 4.0 mg/mL showed cell membrane disruption and nuclear condensation (Fig. 8Bb) as compared to control cells (Fig. 8Ba), while 16.0 mg/mL and 32.0 mg/mL showed significant loss of cellular structure and organelles (Fig. 8Bc,Bd).

#### In vitro antiproliferative activity of Sn-In_2_O_3_ NPs on normal cells (HEK-293)

The effect of Sn-In_2_O_3_ NPs on normal cells (HEK-293) was examined by MTT assay. Post 48 h treatments, we have found that (5%, 10%, and 15%) Sn-In_2_O_3_ NPs did not reduce the cell viability significantly during 48 h of treatment, (details in supplementary file)

## Discussion

At present, the treatment of fungal infections and cancer, are considered among the most important challenges across the world. The problems encountered with the treatment includes, high doses of available drugs and an escalating resistance to these drugs. In this regard, present study demonstrated the successful doping of Sn doped indium oxide NPs (5%, 10%, and 15%) by sonication technique. The results obtained by XRD, SEM, and TEM, proved the favorable doping of tin (Sn) with Indium oxide (In_2_O_3_). Synthesis of different crystal sized NPs, was achieved. It was seen that by increasing the constriction of Sn, the crystallite size also decreased, markedly to be 38.11 nm, 18.46 nm and 10.21 nm, for 5%, 10% and 15%, respectively, which goes in compliance with the reported work of Katsube *et al*. and Ogihara *et al*.^28,29^.

Further, it was observed that the anticandidal activity was significantly enhanced with increased tin content from 5 to 15% over indium oxide. The anticandidal activity was found in the order of Sn doped, 15% >10% > 5% > undoped In_2_O_3_ NPs. This finding is well supported by a previous studies conducted by Martínez *et al*. 2011, who reported the use of indium as dopant for titanium NPs, resulting in the decrease of size of NPs and increase in their antimicrobial properties^30^. There is almost no information available in the literature, regarding the Sn doped In_2_O_3_ NPs against *Candida*. Although, some previous reports about the NPs have examined, that this activity could be associated to size and surface area and the amount of dopant added to the NPs.^31,32^. Moreover, all fungal species including *Candida*, possess a cell membrane, consisting of phospholipids and a cell wall made up of mannoproteins, β-glucan-chitin, β-glucan. Therefore, the NPs need to bind and interact with the macromolecules of cell wall for interaction with the phospholipids, integral and peripheral proteins, and also ionic channels^6,33^. Studies also support the generation of ROS, due to oxidative stress on the microbial cell leading to disruption of cell membrane and cease of cellular activity, which finally leads to cell death^31,34,35^. Presently, the development of new therapeutics for application in biomedicine, like infectious diseases, mainly nosocomial type, and cancer treatment has caused a meaningful interest in the search of nanomaterials with biomedical applications.

Additionally, the cytotoxic impact of Sn-In_2_O_3_ NPs was morphologically and quantitatively evaluated, using human colorectal carcinoma cells (HCT-116). The findings demonstrated that Sn-In_2_O_3_ NPs (5%, 10%, 15%) induced dose dependent manner reduction in cancer cell viability. For example, the lower dosages showed an average 71.10% cell survivability, whereas higher dosages 32.0 mg/mL showed 20.96% reduction in cancer cell viability. In addition, the treatment of Sn-In_2_O_3_ NPs also showed significant cellular and anatomical changes in cancer cells as examined by microscopes. We have used specific colon cancer cell line (HCT-116) to study the impact of different concentrations of Sn-In_2_O_3_ NPs and examined their impact on proliferation of cancerous cells, which we have found that concentration dependent decrease in the cancer cell proliferation as revealed by MTT assay. The prominent changes were which we have observed the disruption of cell membrane, condensation and augmentation of cell nucleus. Moreover, we have also observed significant cell death. There are couple of studies, which have demonstrated cytotoxic effects of NPs, where NPs induced cancer cell membrane disruption, nuclear fragmentation, and disintegration^36,37^. We have also examined the impact of Sn-In_2_O_3_ NPs (5%, 10%, 15%) on normal cells (HEK-293) to explore whether Sn-In_2_O_3_ NPs (5%, 10%, 15%) selectively target the cancerous cells or not. Our results demonstrate that Sn-In_2_O_3_ NPs (5%, 10%, 15%) did not reduce the cell viability of HEK-293 cells (details in supplementary file). Therefore, the results suggested that Sn-In_2_O_3_ NPs (5%, 10%, 15%) possess strong anti-cancer capabilities and could be considered for treatment of cancer.

## Methodology

### Synthesis of Sn doped In_2_O_3_ NPs

Sn doped In_2_O_3_ samples were synthesized through the sonication process. High purity of tin acetate and indium acetylacetonate were obtained from Sigma-Aldrich, and prepared through different dissolved concentrations of the metal precursors (Sn/In = 5%, 10% and 15%) in 36 mL of distilled water. During the sonication process, 4 mL ammonium hydroxide (Sigma-Aldrich) was added slowly to adjust the pH to 9–10. Through direct immersion method, the solution was irradiated for 1 hr via a high intensity ultrasonic processor (750 W; 20 kHz and 6 mm Ti-probe tip). The temperature was increased to 80–90 °C, using a water bath during the sonication system. A homogeneous and transparent solution was obtained. The precipitates were washed and centrifuged number of times with distilled water and later dried at 70 °C. Finally, the products were calcined at 500 °C for 2 h^12^.

### Characterization techniques

The structure and crystallite phase of the Sn doped In_2_O_3_ NPs (Sn/In = 5%, 10% and 15%) samples, were obtained by XRD (Shimadzu XRD-7000, with monochromatic high-intensity Cu Kα radiation (λ = 1.5406 Å)) with 2θ = 14–70 °. The morphology, features, distribution and size of NPs were studied by SEM (Inspect S50) and TEM (Morgagni 268). The sample was prepared for TEM analysis by dispersing in ethanol and shaking in an ultrasonicator for 15 min, and then a suspended drop was dried on the carbon-coated copper grid at room temperature.

### Characterization of anticandidal activity

*C. albicans* ATCC 14053 *was* used to investigate the anticandidal properties of pure In_2_O_3_ NPs and Sn doped In_2_O_3_ NPs (Sn/In = 5%, 10% and 15%). Culture was grown for 24 h in Sabauraud’s broth (SDB) in a shaking incubator (150 rpm) at 28 °C. The obtained cell pellet was washed several times, using phosphate buffered saline (PBS) and finally, cell frequency was adjusted to the concentration of approximately, 10^7^ CFU/ml with sterile 0.9% NaCl.

### Evaluation of MIC/MFC

The MIC of pure In_2_O_3_ NPs and Sn doped In_2_O_3_ NPs (Sn/In = 5%, 10% and 15%) was evaluated by standard agar dilution method. SDA plates were streaked using serial dilutions of the test compound in concentration ranging from 16 to 1 mg/ml, incubated with *C. albicans*. The MIC is defined as the least amount of compound, at which no apparent growth is seen. The lowest concentration of pure In_2_O_3_ NPs and Sn doped In_2_O_3_ NPs (Sn/In = 5%, 10% and 15%), at which no cell was obtained or less than three CFUs was present, was recorded as MFC^38^.

### Effect on candida topography by SEM analysis

The effect of pure In_2_O_3_ NPs and Sn doped In_2_O_3_ NPs (Sn/In = 5%, 10% and 15%) on the topography of *C. albicans* cells were further investigated by SEM. Briefly, 10^6^ CFU/ml cells were treated with 4 mg/ml (conc was selected on the basis of MIC) of pure In_2_O_3_ NPs and Sn doped In_2_O_3_ NPs and incubated at 28 °C for 24 h. Untreated *C. albicans* was also included in the experiment as a control. After incubation, the treated and untreated cells were obtained by centrifugation at 12000 rpm for 10 min. Multiple washing with PBS was done and subsequently, fixed primarily with 2.5% glutaraldehyde, followed by 1% osmium tetroxide for secondary fixation. The fixed samples were again washed several times and dehydrated by 30, 50, 70, 90, 99% ethanol. The cells were placed on the stubs and dried, and gold coated, for examination by SEM, at an accelerating voltage of 20 kV^35^.

### *In vitro* antiproliferative activity of Sn-In_2_O_3_ NPs by MTT assay

#### Cell culture

*In vitro* cell culture was done as per method described by Khan *et al*., 2018 and in brief, human colorectal carcinoma (HCT-116) cells were grown in the media containing DMEM, (5%) L-glutamine, (10%) FBS, (5%) selenium chloride, penicillin and streptomycin. Cells were kept in 5% CO_2_ incubator with 37 °C conditions.

#### Cell morphology

In order to study the impact of Sn doped In_2_O_3_ NPs on cancerous cells morphology, we have grown HCT-116 cells in 6 well culture dishes and when the cells reached to 80% confluency, they were treated with different concentrations of Sn doped In_2_O_3_ NPs. The cells were treated with Sn doped In_2_O_3_ NPs (Sn/In = 5%, 10% and 15%) with these concentrations (2.0 to 32.0 mg/mL). Both control and Sn-In_2_O_3_ NPs-treated cells were analyzed after 48 h intervals. Post 48 h treatment of Sn-In_2_O_3_ NPs, HCT-116 cells were microscopically (TS100F Eclipse, Nikon) examined to evaluate any morphological changes in cells.

#### MTT assay

In order to study the impact of Sn doped In_2_O_3_ NPs on cancer cells proliferation and cell viability, we have done MTT assay as per method described by Khan *et al*., 2018. HCT-116 cells were treated with different concentrations of (2.0 to 32.0 mg/mL) of Sn-In_2_O_3_ NPs. In control group, no Sn-In_2_O_3_ NPs was added. After 48 h of Sn-In_2_O_3_ NPs treatments, the cells were treated with 20 ul of MTT per well and cells were kept in CO_2_ incubator for 4 h. The media was subsequently changed with DMSO and plate were read under ELISA Plate Reader (Biotek- Instruments, USA) at 570 nm wavelength. Cancer cell viability was calculated by this formula:

$$ \% \,\text{of Cell viability}=\text{Optical density}\,(\mathrm{OD})\,\text{of Sn} \mbox{-} {\mathrm{In}}_{2}{{\rm{O}}}_{3}\text{NPs} \mbox{-} \text{treated cells}/(\mathrm{OD})\,\text{Optical density of control cells}\times 100$$

#### Statistical analysis

The mean ± standard deviation (SD) from control and Sn-In203-treated groups were calculated. All statistical analyses were completed with GraphPad Prism 6 (GraphPad Software) and the difference between control and Sn-In203-treated groups were calculated by a one-way ANOVA test (P < 0.05). Where P < 0.05 was considered as statistically significant.

## Conclusion

The obtained results revealed that the doping of Sn (5–15%) over indium oxide was effective enough to enhance the *invitro* biological properties of these nanomaterials. The ability of the NPs, to inhibit the pathogenic *Candida* and cancer cell proliferation, has been demonstrated. The obtained results proved that these materials possess anticandidal and anticancer activities and hold promise for treatment of fungal infections and colon cancer.

## Supplementary information

Supplementary Information.

## Data Availability

The data analyzed are available from the corresponding author upon a request.
