# Supplementary material for: Anticandidal and In vitro Anti-Proliferative Activity of Sonochemically synthesized Indium Tin Oxide Nanoparticles
Source: Sci Rep. 2020 Feb 24;10:3228. doi: 10.1038/s41598-020-60295-w (PMC7040001; doi:10.1038/s41598-020-60295-w)
Supplement: Supplementary file 1 — Supplementary Information. [file 41598_2020_60295_MOESM1_ESM.docx]

**Anticandidal and *In vitro* Anti-Proliferative Activity** **of Sonochemically synthesized Indium Tin Oxide Nanoparticles**

Suriya Rehman^1*^, Sarah Mousa Asiri^2^, Firdos Alam Khan^3^, B. Rabindran Jermy^4^, Vijaya Ravinayagam^4^, Zainab Alsalem^1^, Reem Al Jindan^5^ and Ahsanulhaq Qurashi^6*^

^1^Department of Epidemic Disease Research^,^ Institute for Research & Medical Consultations, (IRMC), Imam Abdulrahman Bin Faisal University, Dammam, 31441, Saudi Arabia.

^2^Department of Biophysics, Institute for Research & Medical Consultations, (IRMC), Imam Abdulrahman Bin Faisal University, Dammam, 31441, Saudi Arabia.

^3^Department of Stem Cell Research, Institute for Research & Medical Consultations, (IRMC), Imam Abdulrahman Bin Faisal University, Dammam, 31441, Saudi Arabia.

^4^Department of Nano-Medicine Research, Institute for Research & Medical Consultations, (IRMC), Imam Abdulrahman Bin Faisal University, Dammam, 31441, Saudi Arabia.

^5^College of medicine, Imam Abdulrahman Bin Faisal University, Dammam, 31441, Saudi Arabia.

^6^Center of Excellence in Nanotechnology, King Fahd University of Petroleum and Minerals Dhahran 31261 Saudi Arabia and Department of Chemistry, Khalifa University of Science and Technology, Main Campus, Abu Dhabi, P.O. Box 127788, United Arab Emirates

*Corresponding authors

Suriya Rehman

E-mail: [surrehman@iau.edu.sa](mailto:surrehman@iau.edu.sa)/suriyamir@gmail.com

[*https://orcid.org/0000-0003-3087-852X*](https://orcid.org/0000-0003-3087-852X)*,*

Phone: 00966532924256

Ahsanulhaq Qurashi

E-mail: ahsan.qurashi@ku.ac.ae

***Methodology***

1. ***Study on hyphal growth of treated C. albicans in liquid medium***

The study was carried out according to the method described by Jalal *et al* 2018 and Ficociello *et al* 2018 with few modification ^1,2^ . Briefly, freshly grown *C. albicans* ATCC 14053 was adjusted to 10^6^ CFU/ml and 2 ml of sterile RPMI 1640 broth (supplemented with sterile pooled sheep serum) having desired concentration of NPs obtained as the MIC in the earlier experiment (8, 4, 2, 1 mg/ml for pure In_2_O_3,_ 5%, 10%, 15% Sn doped In_2_O_3_ NPs, respectively) was added with 10 µl of inoculum and incubated at 37 °C for 3h using orbital shaker. *C. albicans* without NPs was taken as a control. Following the incubation, smears were prepared, visualized and captured using a microscope (Nikon ECLIPSE Ni). The presence of hyphae was manually counted and determined in percentage, corresponding to the total cells per image.

**2.*Biofilm study*** ***of treated Candida by Scanning electron microscopy (SEM).***

*C. albicans* was freshly grown and inoculum was standardized to 10^6^ CFU/ml using sterile normal saline (NaCl 0.9%). Sterile glass cover slips (10 mm in size) were aseptically placed in 12-well tissue culture plate, containing 3 ml of adjusted inoculum of cell suspension in normal saline. For the initial attachment, plate was incubated at 37°C for 1.5 h with gentle shaking at 75 rpm. Normal saline was gently removed and PBS washing was carried out without displacing the glass cover slips, followed by addition of 3 ml of sterile RPMI 1640 broth. Plates were further incubated at 37°C for 48 h for the formation of biofilm. The medium was changed every 24 h. later, the wells were poured with pure and Sn doped In_2_O_3_ NPs in RPMI 1640 at a desired conc, obtained as MIC in an earlier experiment (8, 4, 2, 1 mg/ml for pure In_2_O_3,_ 5%, 10%, 15% Sn doped In_2_O_3_ NPs, respectively). Plates were further incubated for 48 h at 35°C with gentle shaking. Control (untreated) sample was incubated in RPMI 1640 without NPs. Following the incubation, the cover slips were removed and gently washed using PBS and further subjected to fixation in 5% glutaraldehyde for 3 -4 h in a refrigerator. Fixation was followed by dehydration with a series of ethanol solution, and finally air-dried. The fixed samples on cover slips were placed on aluminum stubs using silver paint and coated with gold. The observation was made using SEM at an accelerating voltage of 20 kV ^3,4^.

1. ***In vitro Antiproliferative Activity of Sn-In_2_O_3_ NPs on normal cells (HEK-293)*** ***by MTT Assay***

***Cell Culture:*** *In vitro* cell culture was done as per method described by Khan *et al.,* 2018 for normal cells, human embryonic kidney cells (HEK-293)^5^.

***Results***

***1.Study on hyphal growth of treated C. albicans in liquid medium***

The transformation of yeast to hyphal form is having a pivotal role in the pathogenicity of *Candida ^6^* . Infact, the development of hyphae is crucial for the biofilm formation and thereby for the establishment of infection. Additionally, the formation of germ tubes confers resistance against the immune response like phagocytosis etc ^7^. In the present study, the effects of synthesized NPs on hyphal growth of *C. albicans* was investigated and the obtained results demonstrated that the treated cells were inhibited in terms of growth and the formation of hyphae. The inhibition of hyphal form of *C. albicans* was found approximately 10, 35, 50, 60 and 80% for untreated, pure In_2_O_3,_ 5, 10, 15 % Sn doped In_2_O_3_ NPs, respectively. The hyphae formation was significantly suppressed in 10 and 15 % Sn doped In_2_O_3_ NPs, as compared to the pure In_2_O_3_ NPs, treated and untreated *C*. *albicans* cells (fig 1).


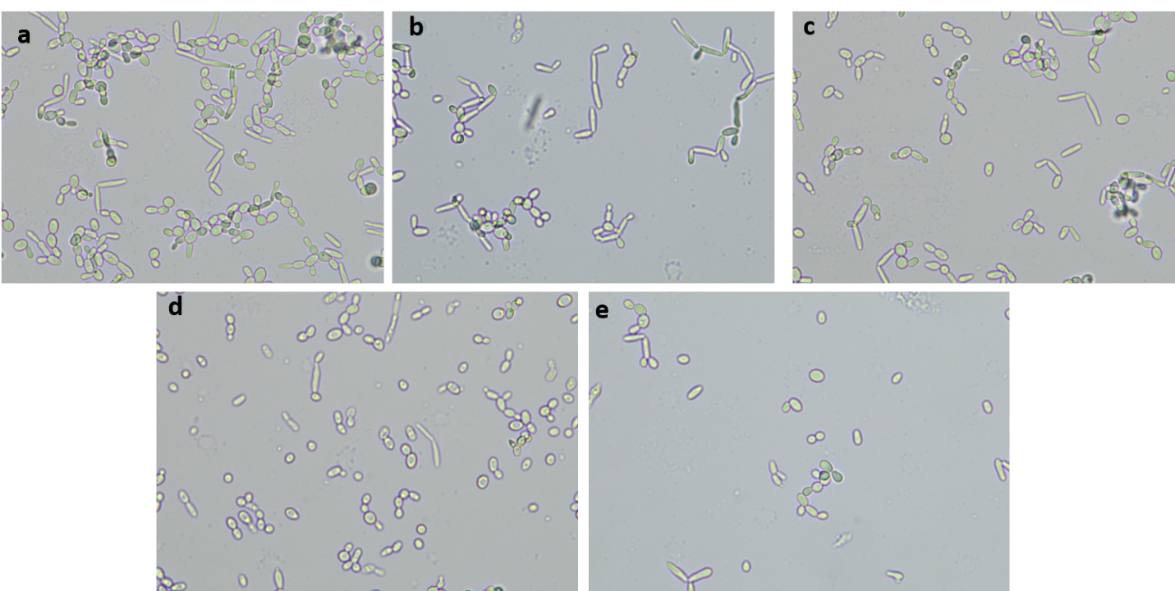


Fig 1.  Inhibition of hyphal formation in *C. albicans* when exposed to. (a) untreated (b) pure In_2_O_3_ NPs (c) 5% (d) 10% (e) 10% Sn doped In_2_O_3_ NPs

1. ***Biofilm study treated Candida by Scanning electron microscopy (SEM).***

SEM was used to study the effect of pure and Sn doped In_2_O_3_ NPs on *Candida* biofilm. Glass cover slips containing biofilms of test *Candida* was exposed to Sn doped In_2_O_3_ NPs (Sn/In = 5%, 10% and 15%) and the untreated *Candida* served as control. Fig 2a shows SEM images biofilm appearance of the *C. albicans* control (untreated) consisting of a dense layer of yeast cells. SEM image of biofilms formed by *C. albicans*, when treated with pure Sn doped In_2_O_3_ NPs produced less-extensive biofilm than the untreated *C. albicans* (Fig. 2b). After exposure to 5%, 10% and 15% Sn doped In_2_O_3_ NPs, biofilms of *C. albicans* exhibited fewer cells and most of which appeared damaged and deformed after the treatment. Biofilms exposed to NPs contained substantially (Fig. 2 c, d & e). the present SEM analysis is an effort to associate the pathogenesis of *Candida* with cellular morphological changes caused by NPs in the biofilm architecture.


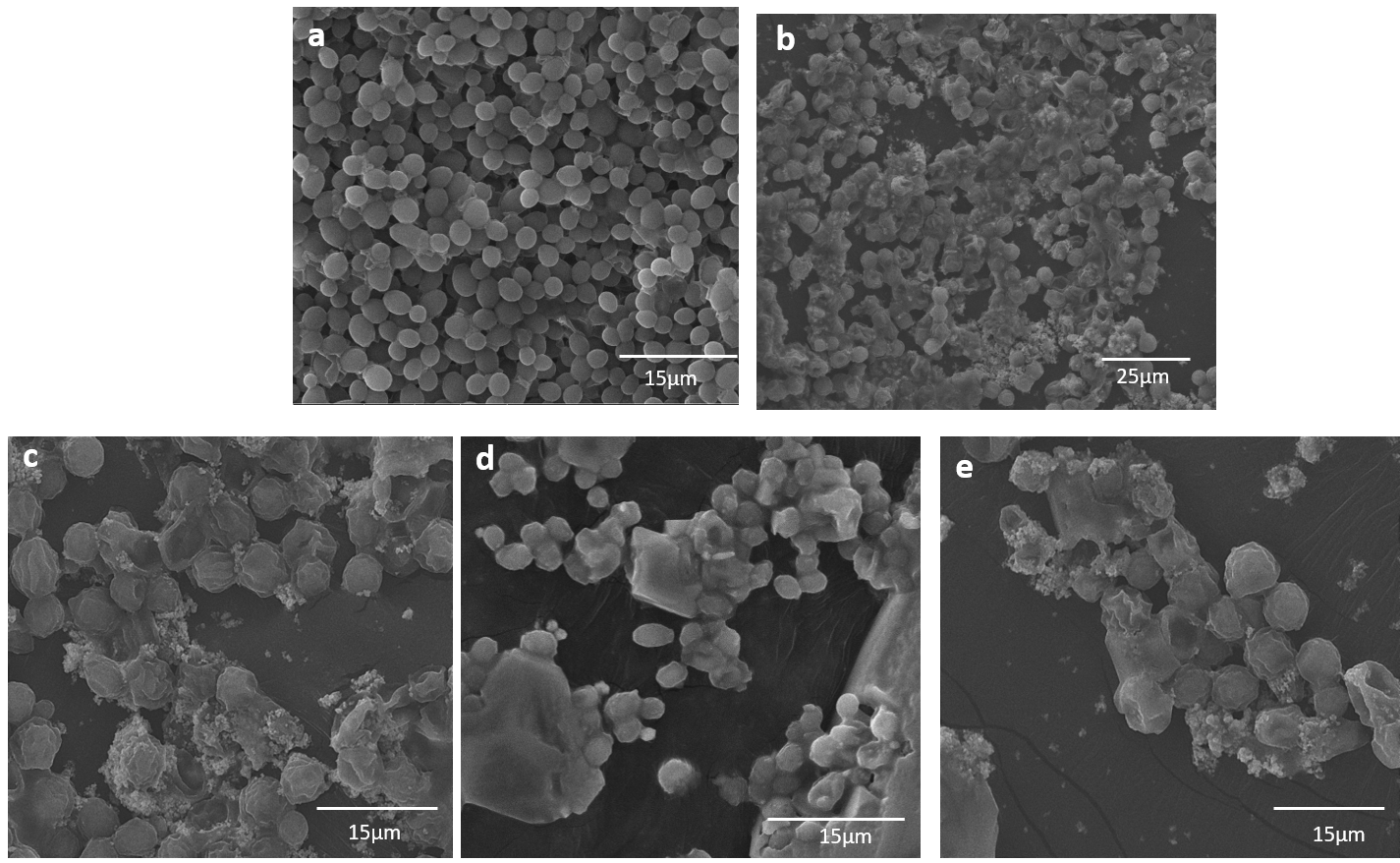


Fig 2.  SEM images *C. albicans* biofilms, when exposed to. (a) untreated (b) pure In_2_O_3_ NPs (c) 5% (d) 10% (e) 15% Sn doped In_2_O_3_ NPs.

1. ***In vitro Antiproliferative Activity of Sn-In_2_O_3_ NPs on normal cells (HEK-293)*** ***by MTT Assay***

The effect of Sn-In_2_O_3_ NPs on normal cells (HEK-293) was examined by MTT assay. Post 48 h treatments, we have found that (5%, 10%, and 15%) Sn-In_2_O_3_ NPs did not reduce the cell viability significantly during 48 h of treatment (Fig.3).


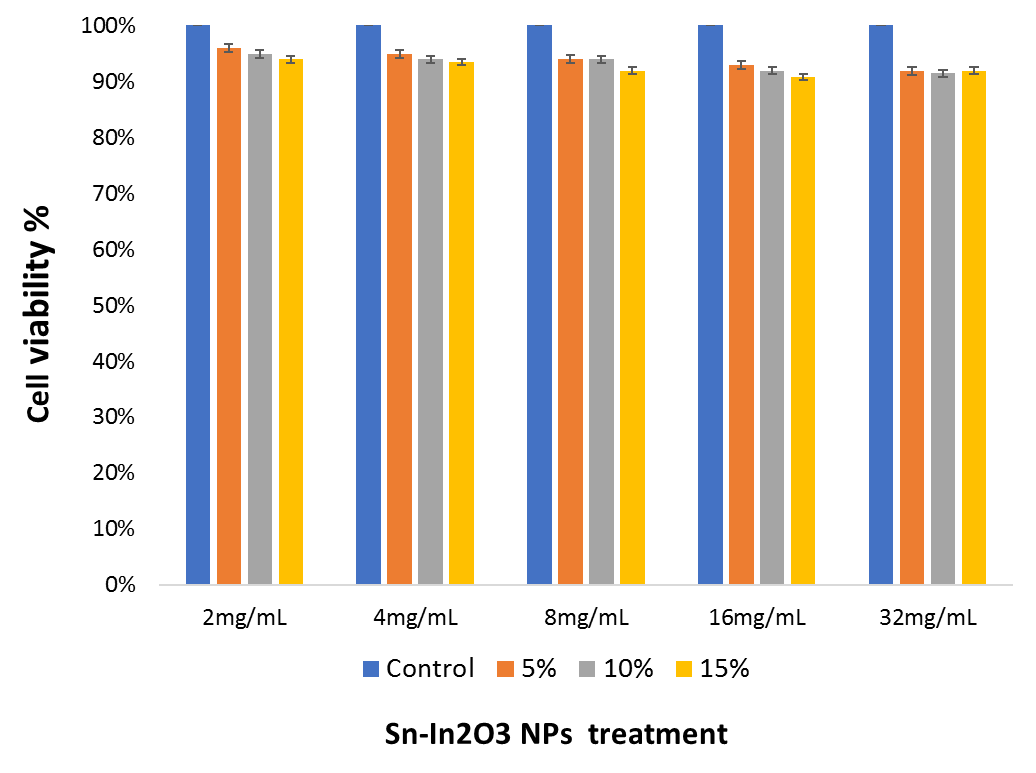


Fig 3. (A) MTT Assay after treatment with different concentrations of Sn doped indium oxide on HEK-293 cells

***References:***

1 Jalal, M., Ansari, M. A., Ali, S. G., Khan, H. M. & Rehman, S. Anticandidal activity of bioinspired ZnO NPs: effect on growth, cell morphology and key virulence attributes of Candida species. *Artificial cells, nanomedicine, and biotechnology* **46**, 912-925 (2018).

2 Ficociello, G. *et al.* Anti-Candidal Activity and In Vitro Cytotoxicity Assessment of Graphene Nanoplatelets Decorated with Zinc Oxide Nanorods. *Nanomaterials* **8**, 752 (2018).

3 Ferreira, J., Carr, J., Starling, C., De Resende, M. & Donlan, R. Biofilm formation and effect of caspofungin on biofilm structure of Candida species bloodstream isolates. *Antimicrobial agents and chemotherapy* **53**, 4377-4384 (2009).

4 Jesus, D. *et al.* Persea americana glycolic extract: In vitro study of antimicrobial activity against candida albicans biofilm and cytotoxicity evaluation. *The Scientific World Journal* **2015** (2015).

5 Khan, S. A., Noreen, F., Kanwal, S., Iqbal, A. & Hussain, G. Green synthesis of ZnO and Cu-doped ZnO nanoparticles from leaf extracts of Abutilon indicum, Clerodendrum infortunatum, Clerodendrum inerme and investigation of their biological and photocatalytic activities. *Materials Science and Engineering: C* **82**, 46-59 (2018).

6 Gow, N. A., Brown, A. J. & Odds, F. C. Fungal morphogenesis and host invasion. *Current opinion in microbiology* **5**, 366-371 (2002).

7 Tronchin, G., Bouchara, J., Robert, R. & Senet, J. Adherence of Candida albicans germ tubes to plastic: ultrastructural and molecular studies of fibrillar adhesins. *Infection and immunity* **56**, 1987-1993 (1988).
